# VR-based training of craniotomy for intracranial aneurysm surgery

**DOI:** 10.1007/s11548-021-02538-3

**Published:** 2021-12-20

**Authors:** Mareen Allgaier, Amir Amini, Belal Neyazi, I. Erol Sandalcioglu, Bernhard Preim, Sylvia Saalfeld

**Affiliations:** 1 Faculty of Computer Science, Otto-von-Guericke University Magdeburg, Universitätsplatz 2, 39106 Magdeburg, Germany; 2 University Hospital Magdeburg, Leipziger Str. 44, 39120 Magdeburg, Germany; 3 Forschungscampus STIMULATE, Magdeburg, Germany

**Keywords:** Virtual reality, Craniotomy, Intracranial aneurysm, Surgical training

## Abstract

**Purpose:**

Intracranial aneurysms can be treated micro-surgically. This procedure involves an appropriate head position of the patient and a proper craniotomy. These steps enable a proper access, facilitating the subsequent steps. To train the access planning process, we propose a VR-based training system.

**Method:**

We designed and implemented an immersive VR access simulation, where the user is surrounded by a virtual operating room, including medical equipment and virtual staff. The patient’s head can be positioned via hand rotation and an arbitrary craniotomy contour can be drawn. The chosen access can be evaluated by exposing the aneurysm using a microscopic view.

**Results:**

The evaluation of the simulation took place in three stages: testing the simulation using the think-aloud method, conducting a survey and examining the precision of drawing the contour. Although there are differences between the virtual interactions and their counterparts in reality, the participants liked the immersion and felt present in the operating room. The calculated surface dice similarity coefficient, Hausdorff distance and feedback of the participants show that the difficulty of drawing the craniotomy is appropriate.

**Conclusion:**

The presented training simulation for head positioning and access planning benefits from the immersive environment. Thus, it is an appropriate training for novice neurosurgeons and medical students with the goal to improve anatomical understanding and to become aware of the importance of the right craniotomy hole.

**Supplementary Information:**

The online version supplementary material available at 10.1007/s11548-021-02538-3.

## Introduction

Intracranial aneurysms (IAs) are pathological dilatations of intracranial vessel walls. A rupture of these dilatations results in a critical bleeding with often fatal consequences. IAs can be treated minimally-invasively or via micro-surgical clipping. Since the last decades, more and more IAs are treated minimally-invasively, as this method has several advantages like, e.g. a reduced operation time [11]. Despite this shift of treatment, there are cases that have to be treated surgically due to complex circumstances, e.g. aneurysms located at the middle cerebral artery (MCA) [10]. The decreasing number of clipping procedures has the negative effect that surgeons and trainees gain less practical experience.

In a clipping surgery, the patient’s head is positioned and fixated and a craniotomy is performed. Subsequently, the IA is dissected and exposed such that a clip can be placed at the aneurysm neck. Due to the combination of little practical experience and complex cases, a strong need for improved training possibilities arises. During the last years, several training methods including physical simulations [12], virtual applications [1, 4, 20, 21] and hybrid methods [22], were presented for IA clipping. We implement a virtual reality (VR) training system which has the advantage that surgeons and trainees can easily explore different approaches without destroying models and with the possibility to undo steps. Consequently, this method is less resource-intensive than cadaver or physical simulations. Concerning virtual simulations, VR is a common method to provide realistic and immersive training in surgery and interventional radiology. Furthermore, virtual applications provide better exploration possibilities such as scaling, rotation or semi-transparent rendering. They also benefit from the possibility of saving intermediate steps and to visualise additional information. Another virtual training possibility would be to use augmented reality (AR). In AR, the main benefit is the combination of real models with virtual models, such as displaying a virtual aneurysm on a real skull. We decided against this, as this would also require more resources as the real skull model would be destroyed. Thus, we developed a VR simulation using a head-mounted display (HMD) to exploit the benefit of immersion.

Many existing aneurysm simulations focus on the actual clipping procedure. Only few also include previous steps like opening the fissure, performing a craniotomy and the head alignment. However, the initial steps are the most important ones, as they enable an easy and proper access and thus facilitate the further steps [18]. The correct position can reduce bleeding, but also provides the most relaxing position for the surgeon. Thus, there are many factors influencing the positioning of the head: planned surgical trajectory, position of the surgeon, gravity retraction or drainage as well as measures for avoiding potential position-related complications like air embolism [18]. We focus on the most important factor: the planned surgical trajectory. Based on a given IA, surgeons have to decide on an approach and an appropriate craniotomy. This procedure and the correlations can be trained with such a simulation.

One relevant craniotomy with respect to MCA aneurysms is the pterional craniotomy which is used for the transsylvian approach [5]. The pterion is defined as the anatomical region nearby the temple where the four bones frontal, parietal, temporal and sphenoid meet. For a pterional craniotomy, the patient is positioned supine, which means that body and legs lie straight. Afterwards, the head is positioned and fixated [16]. Additionally to the pterional craniotomy, other approaches such as the lateral supraorbital approach and the minipterional approach can be used.

The described procedure of the specific case served as a basis for our simulation. Nevertheless, further IA locations and approaches can be trained.

## Related work

There are several approaches for training IA clipping, but only few for access planning. Wong et al. [23] proposed a simulation where the cranial bone can be subtracted for planning. Afterwards, the head can be positioned accordingly and a virtual craniotomy can be performed. Alaraj et al. [1] and Gmeiner et al. [4] also included a craniotomy where the user can draw the outline and a bone volume removal is performed. In the hybrid simulation by Vite et al. [22], a urethane skull model is set up with a Mayfield head clamp before intervention. After the fixation, the virtual space is aligned with the physical space via registration of landmarks. With a haptic device, the craniotomy contour was marked on the physical skull and projected on the virtual skull to generate the corresponding hole. The actual craniotomy was performed only once by cutting a hole into the physical model, supervised by an expert.

As immersive VR is not common in IA clipping simulations, the following related work focuses on VR simulations. The search was restricted to simulations in medicine, using the mentioned display and including an immersive virtual operating room (OR). Pulijala et al. [17] performed a study to assess the effect of VR surgery on the self-confidence and knowledge of surgical residents in case of Le Fort I osteotomy, an essential procedure in craniofacial surgery. Thereby, they also have a virtual, immersive 360$^\circ$ OR, which was appreciated by the surgical residents. To get an immersive 360$^\circ$ OR, Huber et al. [7] recorded an artificial scenario during a laparoscopic surgery and included this into a VR training simulation. Thereby, they compared the normal VR laparoscopic simulation with an immersive simulation using an HMD. The participants using a VR HMD needed more time to complete one of the three tasks and made more mistakes compared to the VR simulation without an HMD. However, they were exhilarated by the high level of immersion, including that they rarely thought about other persons present in the real room and that they felt very present in the virtual world. These aspects lead to an increasing attractiveness of the simulation and thus to more frequent use. Because of these benefits of immersion and presence, we developed an immersive VR training application in contrast to the previously presented simulations in the field of IAs.

**Fig. 1:**
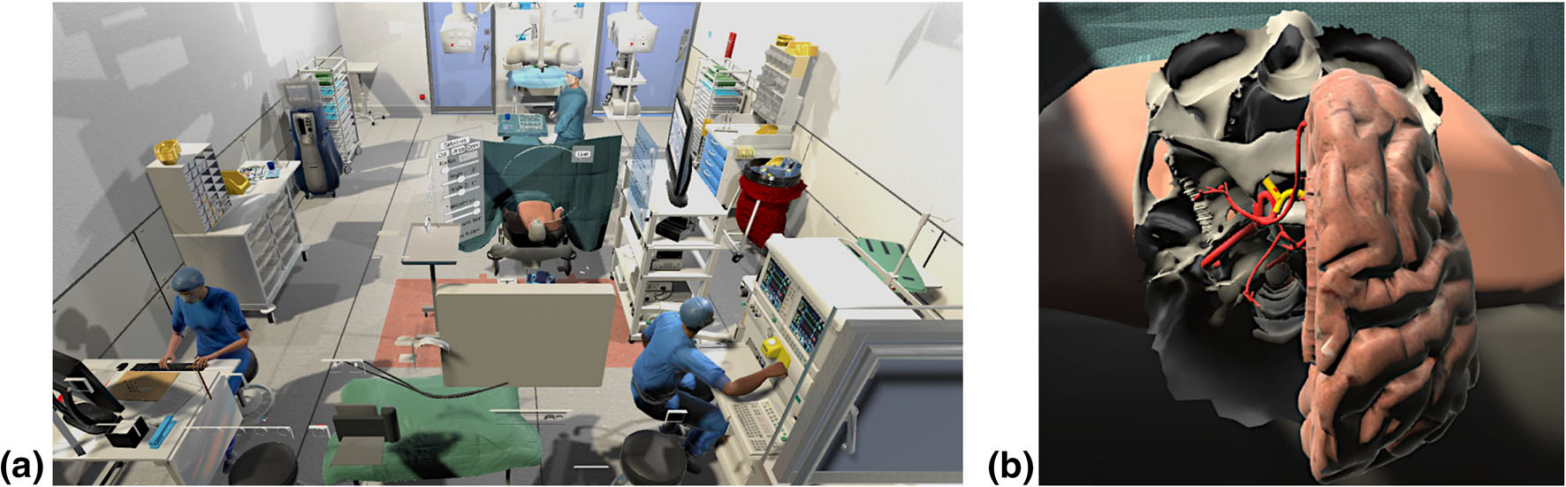
**a** Virtual OR **b** lower part of the skull, right brain half, CoW (red) and optic nerve (yellow)

## Requirement analysis

To develop an effective training and planning simulation, requirements were set up during multiple meetings with a senior neurosurgeon and novice neurosurgeons. An initial meeting served as a basis to gain knowledge about the general procedure and important steps. During the development, several meetings served to optimise the virtual surrounding, the interactions and the models. Based on these discussions, the following requirements were extracted:

*R*1: Head positioning: A head inclination along two axes via intuitive hand rotation should be available. Longitudinal inclination should be possible up to 90 degrees and transversal inclination up to 30 degrees.
*R*2: Craniotomy: The user should be able to draw an individual shape.
*R*3: IAs, suitable for clipping, should be included.
*R*4: For training purposes and improved exploration of vessels and IA, the user should be able to adjust the transparency of skull and brain.
*R*5: Simplification of the anatomical models is feasible, but the following important landmarks should be represented: (a) optical nerve: It serves as an important reference to all other surrounding structures and should be identified early in the exposure [13]. (b) pterion: As this region is a relevant cranial landmark, the sutures of the skull are displayed [18].
*R*6: The virtual environment should be realistic enough to support immersion and not distract the user.
*R*7: Derived from *R*6, it is also important that the user’s posture and position in relation to the patient is similar to those during a real surgery. This mainly affects the transition between the craniotomy and microscopic view.

## Simulation setup

We put the focus on head positioning and craniotomy since these aspects strongly drive the following surgery. In contrast to the above-mentioned simulations that include these steps as well, we decided not to use haptic devices and a stationary stereoscopic display, but to create a more immersive simulation by using a VR HMD and setting up a virtual OR. Consequently, the simulation is not bound to a fixed work station but can be used wherever free space of about $2\times 2$ meters, an appropriate PC or laptop and a VR HMD are available. This is especially advantageous for usage in hospitals.

The simulation was developed with the Unity game engine (Unity Technologies: https://unity.com, San Francisco U.S.) and the XR Interaction Toolkit. During the development, an Oculus Quest and the corresponding controllers were used, but due to the toolkit, transferability to other VR glasses is possible. In our previous work [14] we used an adapted virtual OR similarly to Huber et al. [8], which was significantly expanded in this work. As Fig. 1a) shows, the patient was replaced by an upper body without head, and a segmented and processed spine and skull, including brain and vessels.

### Models

A complete Circle of Willis (CoW) extracted from a healthy person’s MRI data serves as a basis for all IA models. The surface model was segmented with our customised workflow [19]. To meet *R*3, we used the MCA aneurysms from our previous project [2]. Furthermore, the user can also set a small sphere at a desired location at the CoW to approximate a target structure. This enables an easy way to train different situations of various locations of target structures. As mentioned, the predefined MCA bifurcation IAs are based on the same CoW, thus located at the same position not just on the CoW but also in relation to other anatomical structures. Besides the location at the CoW, the characteristics of the specific CoW also matters. Since the length of the M1 segment is an important criterion for the decision about the surgical strategy in MCA aneurysms [3], we provided different CoW configurations for the individual target placement.

The skull is a segmentation of a patient’s computed tomography angiography data that was further processed to reduce artefacts and to simplify the geometry. For simplicity, most of the inner parts of the skull were removed. Additionally, the sutures of the skull were added. As the optic nerve is an important landmark that should be identified after opening the Sylvian Fissure [16], a short fragment of it protruding from the optic canal of the sphenoid bone was modelled. With the optic nerve and the previously described skull model, R5 is met. Furthermore, the sphenoid bone was included to complete this region. Regarding the brain, a free model (https://free3d.com/3d-model/brain-18357.html) was adapted to fit into the skull. The models can be seen in Fig. 1b).

**Fig. 2:**
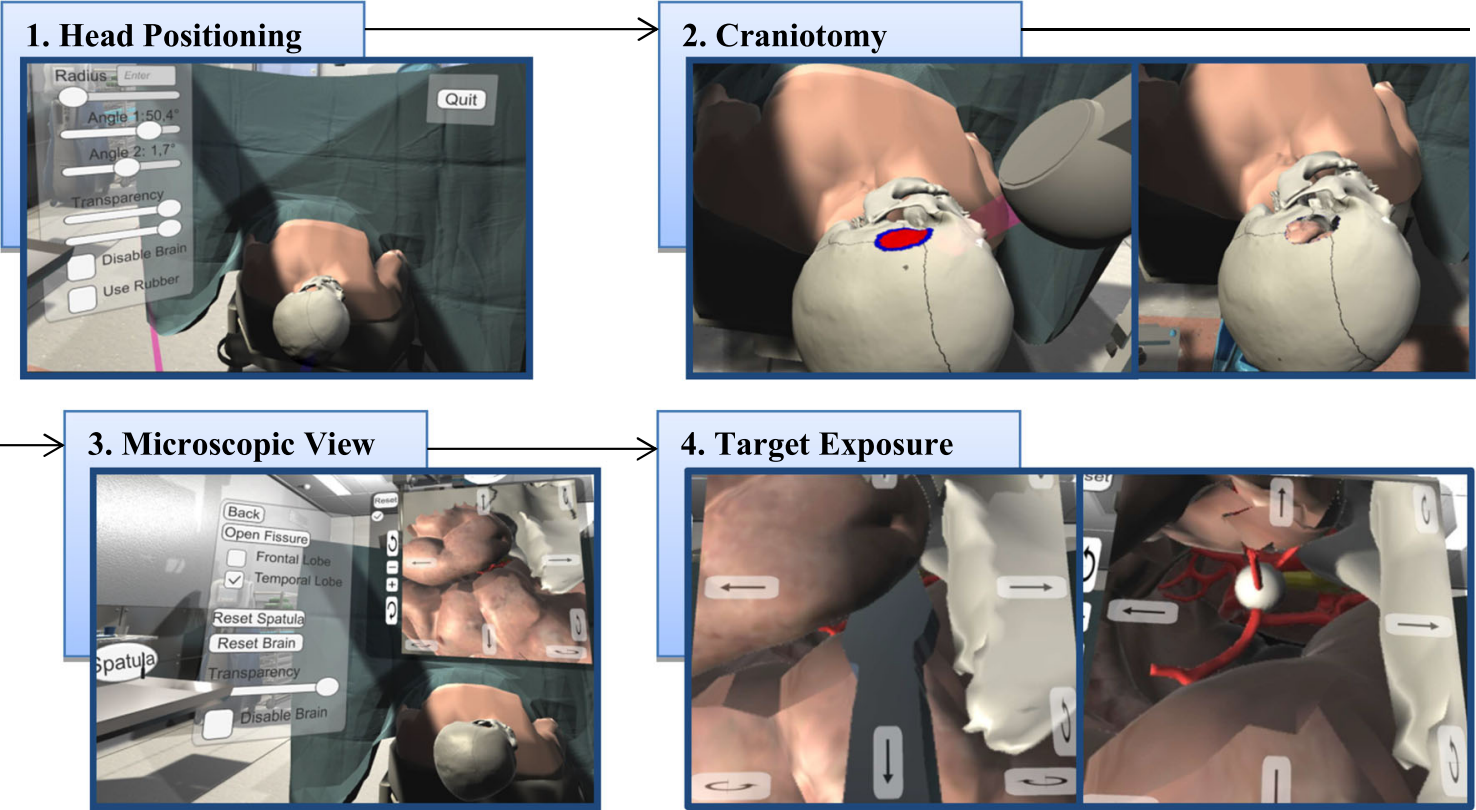
Overview of the simulation tool. 1. Head positioning, 2. Drawing the craniotomy hole, 3. Microscopic view, 4. Opening the Sylvian Fissure to expose the target structure (white sphere) and the optic nerve (yellow structure)

## Workflow

*Head positioning* The first step of the workflow is the head positioning (Fig. 2, step 1). There are two possibilities to rotate the patient’s head. Either via two sliders in the menu or via hand rotation, which is the more intuitive and realistic way (recall *R*1). Therefore, the user has to point at the head and hold the grab button while rotating the hand in the desired direction.

*Craniotomy* By drawing directly on the skull, the users define the contour, size and location of the area, they want to remove (recall *R*2). After defining a seed point in the closed contour, the area is filled by a region growing algorithm (Fig. 2, step 2). Exploring the structures and finding an appropriate location and size of the hole is supported by the adjustable transparency of the skull and brain (recall *R*4).

*Microscopy* After confirming the hole, the simulation switches to the microscopic view (Fig. 2, step 3). When using a microscope during surgery, the surgeon’s viewing direction is straightforward, horizontal to the floor, while the hands are operating at the hole. Thus, they are not looking in the direction of their hands. To simulate this, and thereby realising *R*7, a virtual screen is placed in front of the user and above the patient, displaying the microscopic view. Similar to a real surgery, the microscope camera can be adjusted and moved via an interface next to the display.

*Evaluation* To evaluate the position and size of the hole as well as the trajectory to the IA, the user can open the Sylvian Fissure (Fig. 2, step 4). As the procedure of opening the fissure was not our focus, it is simplified by the following procedure: Taking a spatula and placing it at the fissure. Via the interface, the frontal and temporal lobe can be deformed separately. The underlying deformation calculation is based on our adapted mass-spring deformation using the Verlet integration [2]. The microscopic view and opening of the brain enables the user to evaluate their approach via self-assessment.

## Evaluation

With our simulation we do not aim at replacing training methods but rather at providing an additional training possibility. Thus, we tried to figure out whether the simulation can serve as additional training and whether the expected advantages of using VR HMDs were recognised and appreciated by the participants. To evaluate the mentioned aspects qualitatively and detailed, the think-aloud method was used. Thereby, thoughts and comments of the participant are recorded and in-depth questions can be asked. In addition to this, a small survey served to collect comparative opinions and an overall impression. Finally, the precision of drawing a contour with a controller was assessed.

**Table 1:** Overview of relevant participant’s data.

Age	Gender	Level of experience	Handedness
31–34	Male	Neurosurgeon: 1–5 years	Right
35–40	Male	Neurosurgeon: 11–15 years	Right
26–30	Male	Medical student	Left
21–25	Female	Medical student	Right

### Setup

The evaluation was conducted with four participants with different levels of experience. All relevant data about the participants are summarised in Table 1. It is important to note that one participant used the simulation tool, and VR HMDs in general, for the first time, whereas the others had tried it before. The study was performed separately with each participant. After a short introduction of the tool, the participants were asked to use it on their own. Thereby, they should enter the IA selection, select a CoW and place the target sphere. Subsequently, they were asked to explore the interface and possibilities and to perform a craniotomy. After they were satisfied with the hole, they were requested to set up an appropriate microscopic view to evaluate the craniotomy hole. As during the whole procedure the think-aloud method was used, they were asked to comment on their actions, highlight difficulties and state what they appreciate or what can be improved.

Following was a questionnaire concerning the previous task, which comprised three topics: Immersion, training and load. The topic *immersion* focuses on whether the virtual OR was appreciated and why. As the immersion and presence in the virtual environment depend on the plausibility, the participants should also rate the plausibility of the environment, anatomical models and interactions. The second topic *training* serves to get insights on whether the tool could and would be used for training purposes and in what way it would be helpful: for anatomical understanding, placement of the craniotomy or self-confidence and preparation. The next topic of the questionnaire was the *NASA Task Load Index (TLX)* [6], which is commonly used to assess the load, effort and frustration when solving a task using a software.

The last part served to assess the precision of drawing the craniotomy. To provide an appropriate training system, it should be precise enough to create realistic contours, but also to not frustrate the user. To examine whether a user is able to draw an intended contour, the participants had the task to trace four predefined contours. They are at two different locations with two different line thicknesses each. The templates were drawn by a person with an appropriate medical background and with mouse as input device.

### Results

In the following, the main results obtained by the think-aloud method are summarised and sorted according to the workflow and requirements.

Regarding the *head positioning*, only the participant using the simulation for the first time had difficulties. But after becoming familiar with it, all participants rated it as appropriate. The *craniotomy* was well accepted, but there are two things that should be improved: When using a controller, the hand and arm position is different from the real position. A pen-like device would be more natural. Furthermore, only the outer part of the skull can be removed, but it should be possible to mill the sphenoid bone, too.

All participants liked the additional display for the *microscopic view* and the movement and rotation possibilities. Nevertheless, some suggestions for improvement were mentioned. The interaction with the microscope would be better via joystick or hand rotation instead of user interface buttons. It would also be more realistic if the user has to switch between the two views via a button, because usually it is not possible to see the microscopic view and the patient just by changing the viewing direction.

Concerning the *target exposure*, the participants appreciated the opening of the brain depending on the placement of the spatula and that both brain lobes can be retracted separately. However, two spatulas, different sizes, and a Leyla retractor that can be moved via hand movement would be more natural.

Other important suggestions and feedback are:

- Including sounds to create an even more immersive atmosphere.
- Placing a sphere to simulate various IA locations was strongly appreciated.
- Feedback, for example when rotating the head or retracting the brain too much, would be welcome. This could also be in terms of gamification to increase motivation, fun and ambition.
- Some craniotomy contours could be integrated for learning purposes.

**Fig. 3:**
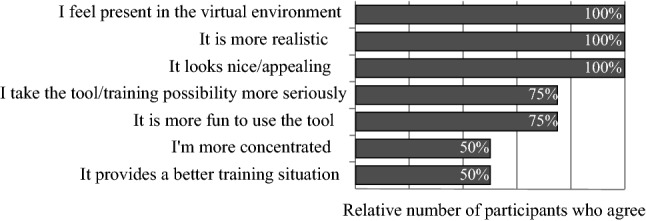
Results of the questions why the participants like the virtual OR in contrast to a simulation with just the head

**Fig. 4:**
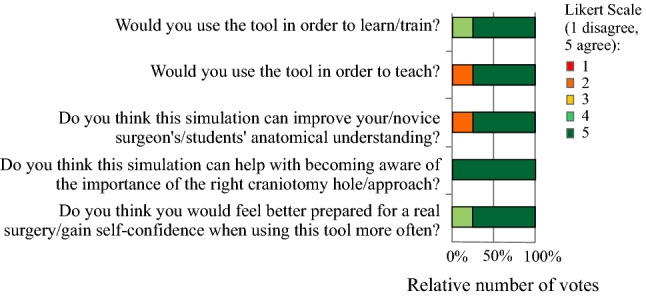
Rating of statements regarding the usage and benefits of the simulation

In the questionnaire we wanted to know whether and why the participants like the virtual environment in contrast to a simulation where only a three-dimensional head is available. The average value using a 5-point Likert scale (0 equals disagreement, 5 equals agreement) of whether they like the OR is 4.5. The reasons why they like it more are displayed in Fig. 3. In Fig. 4, the average ratings of statements concerning the usage and possible benefits of the simulation are shown.

In the NASA TLX questionnaire, a 20-point Likert scale (zero equals very low, 20 equals very high) was used. The results can be summarised as follows. The participants were successful in accomplishing their task ($\varnothing =16.5$, $\sigma =2.06$) and did not have to work that hard to accomplish their level of performance ($\varnothing =8.25$, $\sigma =4.323$). The mental load ($\varnothing =11.5$, $\sigma =4.33$) was rated higher than the physical demand ($\varnothing =9$, $\sigma =5.24$), but both values are in the middle range.

Regarding the craniotomy, we compared the participant’s contour with the template based on the surface dice similarity coefficient (surface DSC) and the Hausdorff distance. The surface DSC indicates the overlap of two contours [15]:

1 $$\begin{aligned} \text {surfaceDSC}(A, B) = \frac{2*\mathrm {TP}}{2*\mathrm {TP}+\mathrm {FP}+\mathrm {FN}} \end{aligned}$$

Additionally, the Hausdorff distance *H*, indicating the proximity of two contours, was also calculated. It is the largest distance of all minimum distances between two curves [9]:

2 $$\begin{aligned} {\begin{matrix} H(A, B) = \mathrm {max}(h(A, B), h(B, A))\\ h(A, B) = \mathrm {max}_{a\in A} \mathrm {min}_{b\in B}||a - b|| \end{matrix}} \end{aligned}$$

**Fig. 5:**
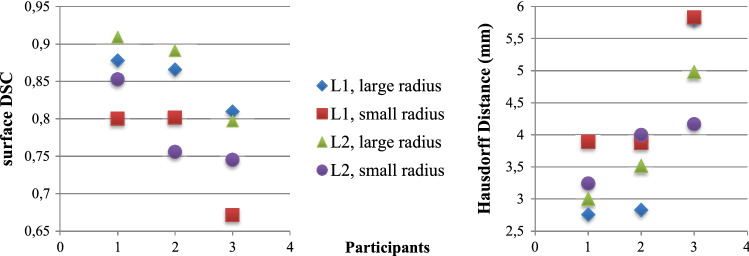
Results of the evaluation with surface DSC (left) and Hausdorff distance (in *mm*; right). L1 and L2 are the two different locations

The average Hausdorff distance is $3.89\, mm$ and the average surface DSC is 0.81.The corresponding results can be seen in Fig. 5. Due to technical problems, the contours of only three participants were available.

## Discussion

During evaluation we observed that the participant using the simulation for the first time and having no previous experience with VR HMDs, had most difficulties. Nevertheless, even in this short time span progress regarding the interactions and drawing a smooth contour could be seen. Thus, using the simulation requires a certain degree of training. Despite of this, the surface DSC and Hausdroff distance results can be rated as precise enough in such a context. The participants mentioned that drawing the contour has a suitable level of difficulty.

With our prototype, we could demonstrate that users appreciate the virtual OR and the resulting realism, immersion and feeling of presence, leading to an increased fun factor and high motivation. Consequently, this kind of simulation offers different advantages than physical simulations, cadaver training or non-immersive virtual training systems and is an appropriate and additional training possibility. These advantages can be further improved by implementing the feedback concerning interactions and the input device, resulting in a more realistic situation.

Regarding our two learning goals of learning the importance of the surgical approach and of improving anatomical understanding, our VR-based training benefits from being non-destructive. Thus, the users can freely explore and try out different approaches with the possibility to undo and redo single steps.

Compared to the IA clipping training approaches presented in “Related work” section, our approach benefits from the immersive environment, whereas the others benefit from the pen-like device and haptic feedback. However, having an appropriate input device is more important during the clipping procedure, where feedback while precisely navigating the clip is essential, than during head positioning and craniotomy. Regardless of the input device, the importance of the right craniotomy placement is demonstrated.

Providing this simulation to surgeons would require gathering feedback from more users, leading to more statistically meaningful results. Nevertheless, the feedback gave detailed insight and serves as a basis for further refinements.

Our simulation comprises realistic anatomical models and consequently provides an appropriate training possibility for neurosurgeons and trainees. In further steps, patient-specific data could be included, resulting in more and various anatomical models, including different access strategies. Despite of the chosen use case of IAs, the simulation could also be used for other surgeries including a craniotomy, like brain tumour removal. With respect to IAs, we could proceed by including further important aspects that were mentioned by the neurosurgeons. Before the craniotomy step, the skin incision and correct opening of the skin can also be part of the training. Regarding the vessel and aneurysm exposure, more details like the dura could be included. Proceeding with the surgery, the clipping process can be implemented, comprising aspects such as the pulse for pulse synchronous clipping. After applying one or multiple clips, there should be the possibility to evaluate the clipping to verify that the aneurysm is sealed off and the normal blood flow in the parent artery is preserved.

## Conclusion

We provided a VR system for craniotomy that benefits from using a HMD, leading to an immersive training possibility where the user feels present in the virtual OR. This simulation can be used for training and teaching with the aim to improve anatomical understanding, to become aware of the importance of the right craniotomy hole and to feel better prepared for real surgery.

## Supplementary Information

Below is the link to the electronic supplementary material.

Supplementary material 1 (mp4 75136 KB)
